# Application of Prostate Resection Endoscopy for Treating Acute Obstruction Associated with Rectal Cancer

**DOI:** 10.7150/jca.69136

**Published:** 2022-03-14

**Authors:** Peng Yan, Yujie Qin, Zhenming Zhang, Wenshan Xu, Jun Qian, Song Tu, Jiaxi Yao

**Affiliations:** 1 Department of General Surgery II, Hexi University Affiliated Zhangye People's Hospital, Zhangye Gansu, 734000, China; 2 Department of Endoscopy Center, Hexi University Affiliated Zhangye People's Hospital, Zhangye Gansu, 734000, China; 3 Department of Urology, Hexi University affiliated Zhangye People's Hospital, Gansu 734000, China; 4 Institute of Urology, Hexi University, Zhangye Gansu, 734000, China

**Keywords:** resectoscope, rectal cancer, obstruction

## Abstract

**Purpose:** To explore a minimally invasive emergency solution for acute obstruction caused by rectal cancer in patients in whom rectal stents or drainage tubes cannot be placed under the guidance of conventional colonoscopy or digital subtraction angiography (DSA).

**Patients and Methods:** Without anesthesia, analgesia, or sedation, the prostate resection endoscopy was inserted into the rectum through the anus, and the rectal space in which the tumor caused obstruction was searched with a certain flushing pressure until it crossed the area of obstruction to reach the proximal intestinal cavity. The drainage catheter or rectal stent was inserted through the sheath of the endoscope to relieve the acute obstruction and permit further cancer treatment.

**Results:** In 31 patients in whom a drainage catheter or rectal stent could not be inserted using conventional colonoscopy or DSA guidance, placement of the catheter or stent into the proximal intestinal cavity was achieved in 28 patients, including drainage tube placement in 21 patients and rectal stent placement in seven patients. Three patients could not undergo placement because of their advanced age and poor general condition. The operative time ranged 15-40 min. Among the 28 patients whose obstruction was relieved, 23 patients underwent radical resection rectal cancer after 10-14 days, and five patients were discharged with stents because they were unwilling to receive further treatment. There were no postoperative complications.

**Conclusion:** Transanal resection is a minimally invasive, effective, safe, and feasible emergency treatment for rectal cancer-associated obstruction.

## Introduction

With the rapid development of society and the economy, the incidence of rectal cancer has increased annually 1, especially in economically underdeveloped areas in northwest of China, where the economic foundation and health awareness are relatively weak 2. Most patients with rectal cancer already have relatively late-stage disease upon presentation, after acute obstruction in particular, the likelihood of colostomy is extremely high, and this greatly reduces the quality of life of patients and increases the difficulty and adverse effects of subsequent cancer treatment. Thus, doctors have been exploring strategies to improve the quality of life of patients with rectal cancer and minimize their trauma. The conventional indwelling stent is placed under colonoscopic or X-ray guidance in the DSA room, but because the colonoscope is long and soft, the directionality and maneuverability are poor. In addition, intestinal contents accumulate in the distal intestinal cavity in patients with hemorrhage and obstruction. When the visual field is unclear, it is difficult to find the gap to insert the guide wire, making it impossible to complete drainage tube or rectal stent placement. Prostate resection using endoscopic technique is the gold standard for surgical treatment of lower urinary tract obstruction caused by benign prostatic hyperplasia. In recent years, our hospital has applied prostate resection endoscopy to relieve acute obstruction associated with rectal cancer. There are rarely any reports in the literature. This study describes our results and accumulated experience.

## Patients and Methods

### General information

From 2006 to October 2020, 31 patients in our hospital who could not undergo rectal stent or drainage tube placement under the guidance of conventional colonoscopy or digital subtraction angiography (DSA) underwent prostate resection endoscopy to relieve acute obstruction associated with rectal cancer. We included only patients with acute obstruction caused by rectal cancer, lithotomic position can be placed, able to tolerate pain or a small dosage of analgesic and sedative drugs. We exclusion patients with severe cardiopulmonary dysfunction cannot tolerate anesthesia and pain, and their family members refused this type of surgery, rectal rupture, severe anus stricture. The study cohort included 17 men and 14 women aged 45-94 years, 80.6% were over 70 years, the operation time was 15-40min. The duration of obstruction was less than 5 days in 21 patients and more than 5 days in 10 patients. The distance between the lower edge of the tumor and the anal edge was longer than 10 cm in 8 patients, between 5 and 10 cm in 16 patients, and shorter than 5 cm in 7 patients. In the end, rectal stents were successfully placed in 7 patients, and drainage tubes were placed in 21 patients. All patients were treated with antibiotics to prevent infection, and 16 patients required painkiller (Table 1). The study was officially approved by the Ethics Committee of Hexi University Affiliated Zhangye People's Hospital and written informed consent was obtained from each patient.

### Surgical method

**Figure 1:**
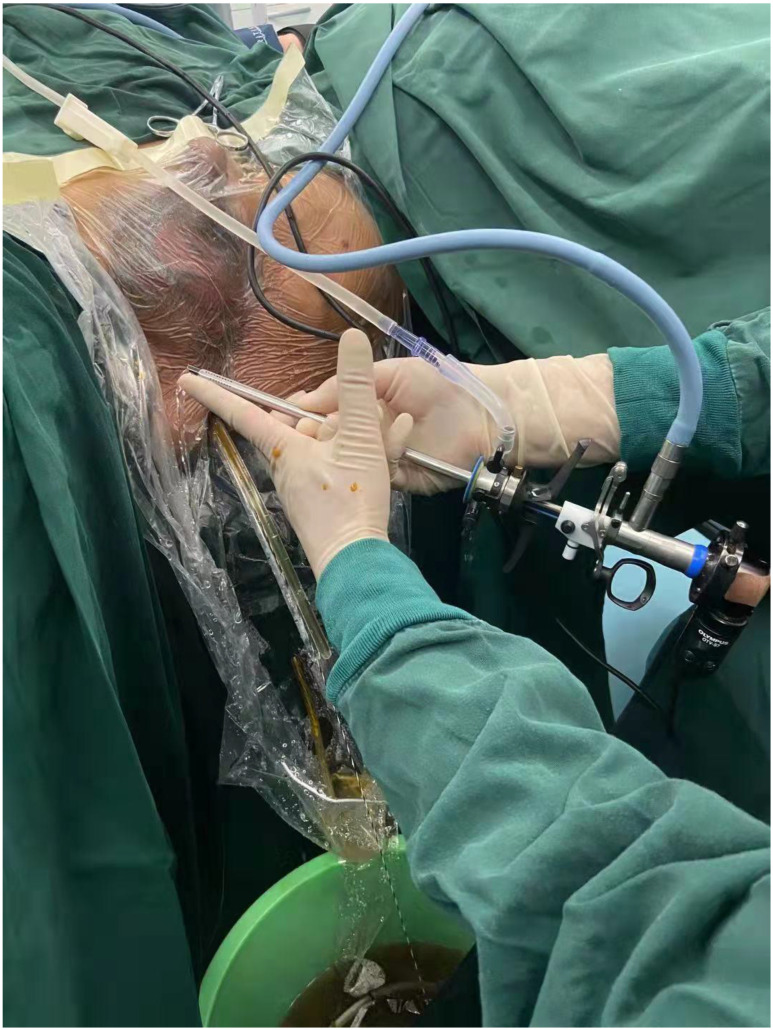
A 26.5-Fr (diameter, approximately 8 mm) Olympus prostate resectoscope was inserted into the rectum through the anus.

**Figure 2:**
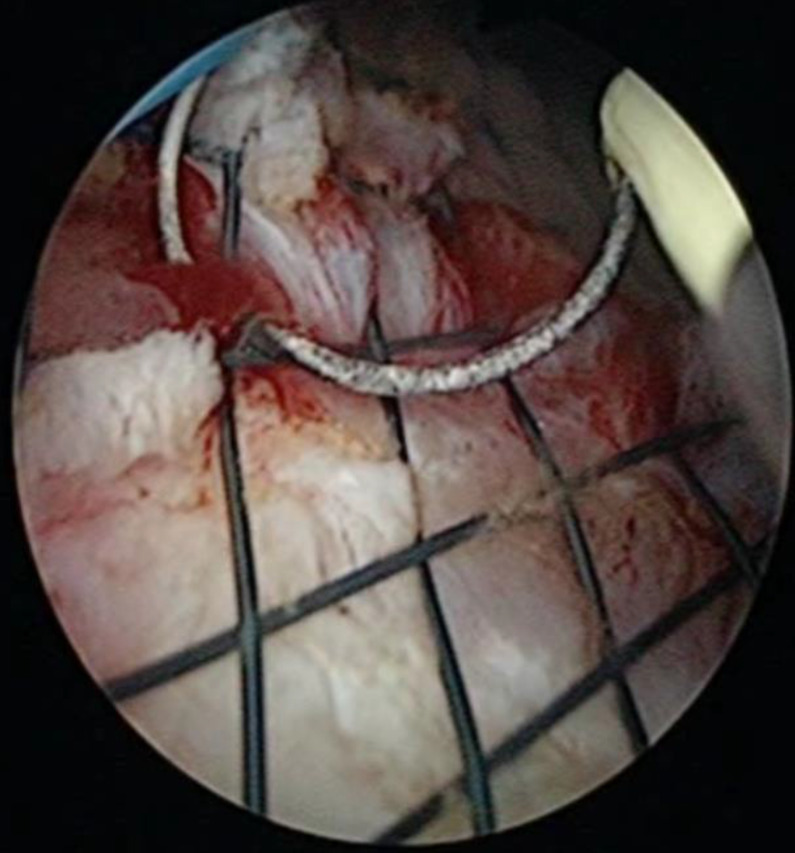
The position of the drainage catheter or rectal stent was appropriately adjusted as needed.

In the surgery, each patient assumed the lithotomy position, and the hip was raised approximately 5 cm to facilitate the procedure. The perineum was then disinfected. Although anesthesia was not used, nervous patients or those with obvious abdominal distension or abdominal pain because of intestinal obstruction were provided analgesics and sedatives. A 26.5-Fr (diameter, approximately 8 mm) Olympus prostate resection endoscopy was inserted into the rectum through the anus (Figure 1). The prostate resection endoscopy has a water inlet and a water outlet for flushing. The inlet is connected with normal saline, and the prostate resection endoscopy slowly enters the rectum. When the tumor is observed, the electrosurgical loop (the electrosurgical “knife” for transurethral resection of the prostate, used for tissue cutting and electrocoagulation) approach for the tumor and look for gaps.The flushing fluid was 0.9% saline, and the flushing pressure was 60-80 cm H_2_O. The anus was observed while flushing. By increasing the flushing pressure, the mirror sheath and electrode gently squeezed the tumor obstruction site to find the internal space of the rectum that was completely obstructed by the tumor. Squeezing, pushing, expanding, and other techniques were used to smoothly reach the proximal intestinal cavity of the obstruction until crossing the obstructive segment. Expansion of the rectum and the accumulation of feces and gas were observed. The outer sheath of the resectoscope was indwelled, the inner sheath and observation mirror were removed, and two guide wires were inserted through the outer sheath of the resectoscope (one safety guide wire and one treatment guide wire). The prostate resection endoscopy was removed and reinserted through the anus. Under the surveillance of the prostate resection endoscopy, the drainage catheter (an F22 balloon catheter was used to prevent detachment of the tube) or rectal stent (6×2.5 cm^2^) was placed with the treatment guide wire into the narrow intestinal canal, and the position of the drainage catheter or rectal stent was appropriately adjusted as needed (Figure 2). The resection scope was withdrawn, and the drainage catheter was fixed with 4-0 silk suture onto the skin next to the anus (1-2 cm from the anal edge).

If it was difficult to find the gap, the spray mode was used with 280 W of cutting power and 160 W of coagulation power, and the ring electrode was used to remove part of the tumor tissue from the suspicious gap in the tumor center (the removed tumor tissue was submitted for pathological examination) to find the gap, and the surgical wound was electrocoagulated to stop the bleeding (Video 1) in supplementary material. The tumor tissue was removed, and lavage fluid was discharged during the operation to maintain a low-pressure state in the rectum, thereby reducing water absorption and preventing water intoxication.

## Results

In this group of 31 patients with obstruction of the intrarectal space, the proximal intestinal cavity of the obstruction was successfully reached in 28 patients, and the rectal obstruction was immediately relieved, three patients could not undergo placement because of their advanced age and poor general condition. During the operation, intestinal gas and feces were elicited from the endoscopic sheath. Most patients expressed that their abdominal distension was reduced during the surgery. An indwelling drainage tube was inserted into 21 patients, whereas 7 patients underwent rectal stent placement. The intrarectal space that was completely obstructed by the tumor was difficult to locate in three patients, and the treatment was discontinued because of their age and inability to tolerate the procedure. The median operative time was 21 min. Of the 28 patients whose obstruction was resolved, 23 underwent radical resection or resection of the rectal tumor after 10-14 days, and 5 patients were discharged with rectal stents after refusing further treatment. No patients experienced significant pain during the surgery, and no complications such as intestinal perforation, significant bleeding, severe infection, and anal sphincter injury were observed after the procedure. Patients are routinely treated with antibiotics before and after operation, the stents and drainage tubes in the intestine are taken out after second stage operation.

## Discussion

Colorectal cancer is the second leading cause of cancer death in western European countries 3. Colorectal cancer is the third most common malignant tumor in China and the fifth leading cause of cancer death 2. Compared with the incidence in Europe and the US, the incidence of colorectal cancer is higher in China 4, and thus, the incidence of intestinal obstruction caused by rectal cancer may be higher 5,6.

Acute obstruction is a serious complication of advanced rectal cancer, and its incidence is approximately 9.3%-15.0%. Approximately 20% of patients with colorectal cancer will require emergency surgery, and the surgical mortality rate is approximately 20% 7. Previously, the surgical methods used for acute obstruction caused by rectal cancer mainly included primary resection and anastomosis, the Hartmann procedure, three-stage surgery (primary colostomy, secondary tumor resection, and tertiary closed stoma), and colostomy 5,6. Patients requiring emergency surgery generally have a poor condition, including water-electrolyte and acid-base balance disorders, anemia, hypoproteinemia, hypertension, diabetes, and cardiovascular and cerebrovascular diseases. The mortality rates of primary resection and anastomosis and rates of postoperative complications such as intestinal fistula, abdominal cavity infection, sepsis, septic shock, and incision infection are high. The Hartmann operation involves colostomy. After the operation, some patients must undergo a second stoma creation according to their physical condition. Multiple surgeries increase the risk of complications such as tumor implantation, spread, intestinal fistula, and incision infection^7^. Meanwhile, patients with a poor physical condition such as older patients may be unable to undergo repeat stoma creation. Colostomy and the Hartmann procedure are limited by the fact that the artificial anus decreases patients' quality of life poor and increases the psychological burden and risks of related complications, such as skin erosion, fistula retraction, and stenosis 8. Bogdan et al. reported postoperative complications following surgery for rectal cancer. The anastomotic leak rate, reported was 11%, the pelvic sepsis rate was 12%, the postoperative death rate was 2% and the wound infection rate was 7% 9.

The American Society of Colorectal Surgeons' Rectal Cancer Guidelines state that intestinal stents can be used palliatively to relieve obstruction in patients with colon or rectal obstruction 10,11. Treatment under DSA guidance is relatively blind, which easily leads to complications such as intestinal perforation and bleeding. Consequently, doctors can only perform emergency colostomy, emergency tumor resection and colostomy, or emergency tumor resection and colorectal anastomosis 12. However, these three types of emergency surgery do treat the tumor or preserve anal function, and emergency colorectal anastomosis carries high risks of infection, intestinal fistula, and other operations. Reports in the literature indicate that the success rate of stent placement early success was 73.3% 13. In our experience, the success rate of indwelling drainage tube or stent placement under colonoscopy less than 50%, and the rate remains low even under DSA guidance. This low rate may be associated with the high rate of advanced obstructive rectal cancer in western China.

In 2006, we developed a transanal resectoscopic technique to relieve complete obstruction caused by rectal cancer. Through 15 years of treatment and follow-up, we have accumulated substantial experience. The evidence indicates that transanal resection is simple and effective for treating acute obstruction caused by rectal cancer. The treatment can be performed under non-anesthetic conditions. Even if the patient is nervous and intestinal obstruction, abdominal distension and abdominal pain are obvious, only analgesics and sedatives are needed. The completion of surgical treatment has the advantage of minimally invasive, and even if it is unsuccessful, there is almost no harm to the patient. Prostate resectoscopy has many advantages over commonly used colonoscopes. First, it has a rigid lens with orientation marks and good directionality. Second, the lens body is short and easy to operate. Third, the operating channel is large, and the sheath can pass through an 8-mm (F24) catheter. Fourth, the flushing fluid has a high flushing pressure when the resectoscope works, making it easy to find the gap near the tumor. The sheath can act as a dilator when necessary. Fifth, the diameter of the resectoscope sheath is 8 mm, making it easy to pass through narrow gaps, whereas a colonoscope generally has a diameter of 12-14 mm. The resistance and damage are greater. In addition, because of continuous washing, the intestinal cavity at the distal end of the site of rectal obstruction is washed, providing clear vision and making it easy to find the gap. Under the microscope, guide wire catheter insertion, tumor resection, electrocoagulation, and hemostasis can be performed. Once the drainage tube or rectal stent is successfully placed, the rectal obstruction can be quickly relieved in patients with resectable tumors, the pressure of the intestines and edema of the intestinal wall can be reduced, and the general condition of the patient can be improved. Once the bowel preparation is completed, radical surgery can be performed within a limited time. This strategy increases the likelihood that patients will choose further reasonable and safe surgical methods (such as Dixon surgery). If the lesion is unresectable or the patient has contraindications to surgery, an indwelling stent or drainage tube can be used to relieve the intestinal obstruction, replace the traditional palliative colostomy, and improve his or her quality of life.

To treat acute obstruction attributable to rectal cancer, a diversified comprehensive treatment model must be adopted according to the patient's physical condition and tumor biological characteristics. It requires the collaboration of multiple physicians, but the basic principle is same; that is, the obstruction should be corrected first before curative treatment is considered. However, there are some limitations in this study, this procedure only relieved the obstruction, but cannot eliminate the tumor, and the patient needs another operation to resect the tumor. Although it reduces the risk of surgery, it also prolongs the time to next surgery.The number of clinical cases was small, and it is necessary to further accumulate and summarize our experience. In addition, prospective clinical controlled studies are required.

Prostate resection is a basic technique performed in China primary hospitals 14. Urology specialists or part-time general surgery doctors can easily master the technique of prostate resection and use the technique in the treatment of rectal diseases. In addition, prostate resectoscope technology can be used to treat early-stage rectal and sigmoid tumors and bleeding within 20 cm of the anal edge (scope length is 20 cm), this technique is especially suitable for tumors and bleeding below the peritoneal reflex (within 8 cm of the anal edge). The resectoscopic technique is easier to master than transanal endoscopic microsurgery, and the technique is additionally associated with a shorter learning curve and lower equipment cost. Moreover, it can be applied in multiple disciplines. If stent placement cannot be completed under colonoscopic or X-ray guidance, the transanal resectoscope technique is an alternative option that could facilitate further treatment. This technology can serve a large number of patients with advanced rectal cancer-associated obstruction in poor areas of developing countries.

## Supplementary Material

Supplementary video 1.

## Figures and Tables

**Table 1:** Clinical characteristics of patients

Characteristics	Patients(n)	%
No. of patients	31	100
Gender		
Male	17	54.8
Female	14	45.2
Age		
≤70 years	6	19.4
>70years	25	80.6
Intestinal obstruction time		
≤5 days	21	67.7
>5 days	10	32.3
Relief of obstruction plan		
Stent placement	7	22.6
Drainage tube placement	21	67.7
Unsuccessful	3	9.7
Operation time (min)	15-40	
Distance between the lower edge of the tumor and the anal edge		
≤5 cm	7	22.6
>5 and ≤10 cm	16	51.6
>10 cm	8	25.8
Postoperative medication		
Antibiotic	31	100
Painkiller	16	51.6
